# Engineering an endocrine Neo-Pancreas by repopulation of a decellularized rat pancreas with islets of Langerhans

**DOI:** 10.1038/srep41777

**Published:** 2017-02-02

**Authors:** H. Napierala, K.-H. Hillebrandt, N. Haep, P. Tang, M. Tintemann, J. Gassner, M. Noesser, H. Everwien, N. Seiffert, M. Kluge, E. Teegen, D. Polenz, S. Lippert, D. Geisel, A. Reutzel Selke, N. Raschzok, A. Andreou, J. Pratschke, I. M. Sauer, B. Struecker

**Affiliations:** 1 Department of Surgery, Campus Charité Mitte and Campus Virchow Klinikum, Charité - Universitätsmedizin Berlin, Germany; 2 Department of Radiolgoy, Charité – Universitätsmedizin Berlin, Germany; 3 Berlin Institute of Health (BIH), Berlin, Germany

## Abstract

Decellularization of pancreata and repopulation of these non-immunogenic matrices with islets and endothelial cells could provide transplantable, endocrine Neo- Pancreata. In this study, rat pancreata were perfusion decellularized and repopulated with intact islets, comparing three perfusion routes (Artery, Portal Vein, Pancreatic Duct). Decellularization effectively removed all cellular components but conserved the pancreas specific extracellular matrix. Digital subtraction angiography of the matrices showed a conserved integrity of the decellularized vascular system but a contrast emersion into the parenchyma via the decellularized pancreatic duct. Islets infused via the pancreatic duct leaked from the ductular system into the peri-ductular decellularized space despite their magnitude. TUNEL staining and Glucose stimulated insulin secretion revealed that islets were viable and functional after the process. We present the first available protocol for perfusion decellularization of rat pancreata via three different perfusion routes. Furthermore, we provide first proof-of-concept for the repopulation of the decellularized rat pancreata with functional islets of Langerhans. The presented technique can serve as a bioengineering platform to generate implantable and functional endocrine Neo-Pancreata.

Type 1 diabetes mellitus (T1DM) is one of the most cost-intensive chronic diseases worldwide^1^,^2^. Insulin was discovered more than 90 years ago and has since been the main therapeutic agent in the management of T1DM; however, in some cases, exogenous insulin replacement is not able to provide the necessary metabolic regulation to prevent primary (hypoglycemic episodes)^3^ and secondary long-term complications (e.g., vasculopathy, cardiovascular diseases, diabetic nephropathy, neuropathy and retinopathy)^4^. In these severe cases, Β-cell replacement through pancreas or islet transplantation may be indicated.

Pancreas transplantation is a well-established procedure and (with regard to long term metabolic function) superior to islet transplantation^5^. However, in addition to rejection, major and occasionally life-threatening complications, such as post- transplant-pancreatitis, infections and thrombosis, continue to result in approximately 10% graft loss. Islet transplantation appears to be an alternative because it is less invasive and is therefore considered safer^6^. Although the indications to islet (auto-) transplantations are currently being expanded^7^, metabolic function after islet transplantation continues to suffer from various issues, such as the need for immunosuppression and graft-site specific problems like hypoxia^8^, partial portal vein thrombosis^9^,^10^ and the instant blood-mediated inflammatory reaction (IBMIR)^11^.

With the development of novel tissue engineering techniques (e.g., decellularization and recellularization; Fig. 1), the above-mentioned issues could be overcome^12^. The intent of decellularization is to remove all cellular and antigenic material from an organ while preserving the innate and possibly non-immunogeneic, extracellular matrix (ECM). This matrix could be used for the bioengineering of transplantable organs via repopulation with (autologous) cells^13^,^14^,^15^,^16^. The ECM represents a biochemically, geometrically and spatially ideal platform^17^, preserves basic matrix components, such as proteins and growth factors^18^, and retains an intact vasculature^17^. This environment could be the key to improved long-term islet survival and function after transplantation^6^.

Furthermore, by applying this technique it appears possible to engineer a solely endocrine Neo-Pancreas, which would prevent post-transplant pancreatitis because exocrine cells would not be used for the repopulation of the organ. The need for post-transplant immunosuppression could in the future be avoided, given that a protocol for the differentiation of fibroblasts into beta-like cells is already available^19^.

However, surprisingly, a limited number of studies have been published reporting on pancreas decellularization and recellularization^1^,^20^,^21^,^22^,^23^ compared with other organs. To the best of our knowledge, no study is currently available reporting on whole rat pancreas decellularization, although a decellularized rat pancreas would render an interesting platform for tissue engineering experiments. Furthermore, no data is available on the repopulation of decellularized pancreas matrices with intact islets. Thus, it remains unclear, if the decellularized, parenchymal space can be repopulated with whole, intact islets, which are significantly bigger than other cells^24^.

Our aim was to find a rapid and reproducible protocol to decellularize whole rat pancreata and repopulate these matrices with intact Islets of Langerhans. Pancreas perfusion is possible through various perfusion routes (portal vein, aorta, extrahepatic bile duct and consecutively the pancreatic duct); thus, we evaluated these routes regarding decellularization effectiveness, ECM conservation, general handling and the possibility to infuse whole islets into the matrix. Furthermore, we evaluated if islets are still viable and functional after the process by *ex vivo* perfusion and glucose stimulated insulin secretion of repopulated Neo pancreata.

## Results

### Macroscopic observations during decellularization

**Figure 2:**
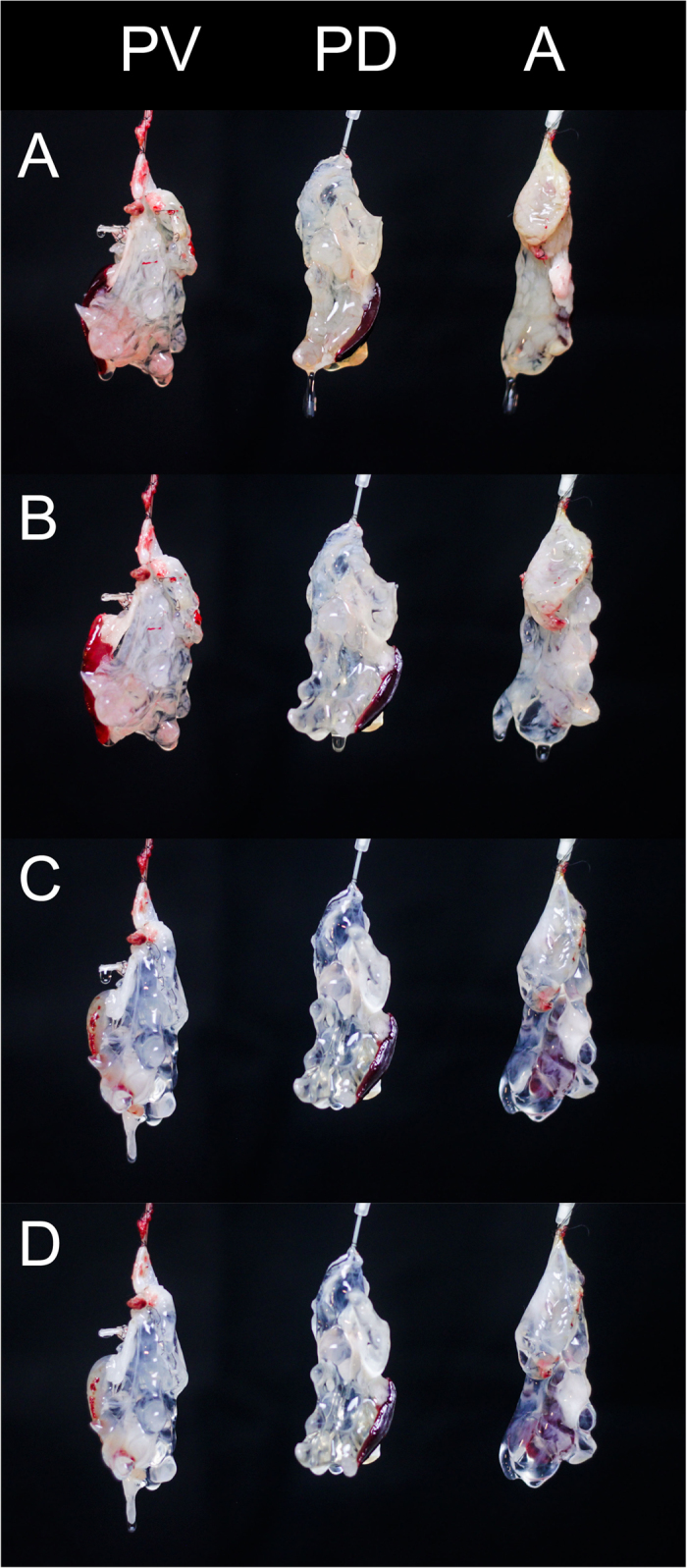
Macroscopic observations during decellularization. Time course of perfusion of three rat pancreata cannulated via the portal vein (PV), the pancreatic duct (PD) or the arterial system (A). At the initiation of the perfusion pancreata appear yellow to brown. Attached to the pancreata is the spleen in dark brown. One hour after perfusion with Triton X-100 pancreata start to lose their color and elucidate. After 2 hours of perfusion with SDS the pancreata appear completely transparent. The spleen is still brown because splenic blood vessels were ligated and not perfused. At the end of the decellularization process the organ structure is still conserved but the cellular components are removed.

During perfusion decellularization, a gradual loss of brownish color could be observed in all three groups until the organs appeared lucent (Fig. 2A–D).

After 1 hour (initial perfusion with Triton X-100) the organs remained opaque but elucidated compared with the beginning of the experiment (Fig. 2B). After 3 hours (end-point of the perfusion with SDS), the organs were completely translucent (Fig. 2C). This did not change until the end of the protocol (Fig. 2D). Macroscopically, the generated scaffolds were completely cell free but remained an intact network of vessel-like-structures.

### Histological evaluation of decellularized matrices

**Figure 3:**
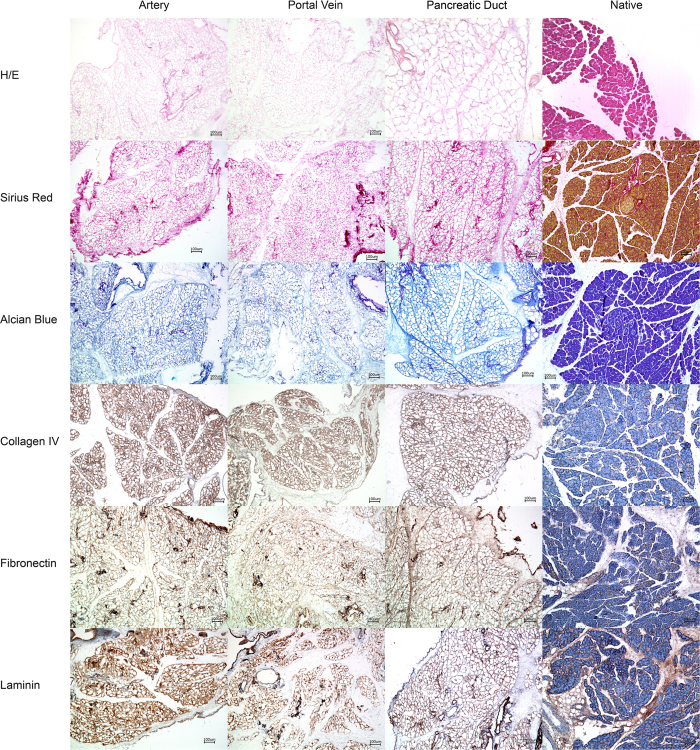
Histology of the decellularized pancreata. Pancreata that were decellularized via the Artery, the Portal Vein or the Pancreatic Duct were stained with different methods to analyze the organ structure after decellularization and compared with native controls: No remaining cells were found inside the decellularized organs but the characteristic lobular microarchitecture of the organ was preserved. Key matrix proteins (Collagen IV, Fibronectin and Laminin) were visualized by immunohistochemical stainings. No significant differences were observed between the three experimental groups. Key matrix proteins remained in the matrices.

Macroscopic observations were confirmed by H/E staining (Fig. 3): no cellular material was stained in the decellularized matrix, whereas the lobular structure of the pancreas could be preserved. Additionally, the connective tissue septa, as well as the ductal and vascular network were preserved in all specimens. Sirius red and Alcian Blue stainings were performed to visualize collagen fibers and acidic sulfated mucopolysaccharides as abundant components of the ECM, respectively (Fig. 3). The decellularized ECM appeared identical to that of the controls in all cases. Microscopically, no relevant differences were observed between the three experimental groups (Fig. 3).

Immunohistochemical stainings were performed to visualize collagen IV, fibronectin and laminin as abundant components of the ECM for a qualitative evaluation of the ECM after decellularization compared with the controls (Fig. 3). In all cases, the matrix proteins were detected inside the matrix and their distribution appeared comparable to native tissue. Again, no relevant differences were observed between the three experimental groups (Fig. 3).

### Biochemical analysis of decellularized matrices

**Figure 4:**
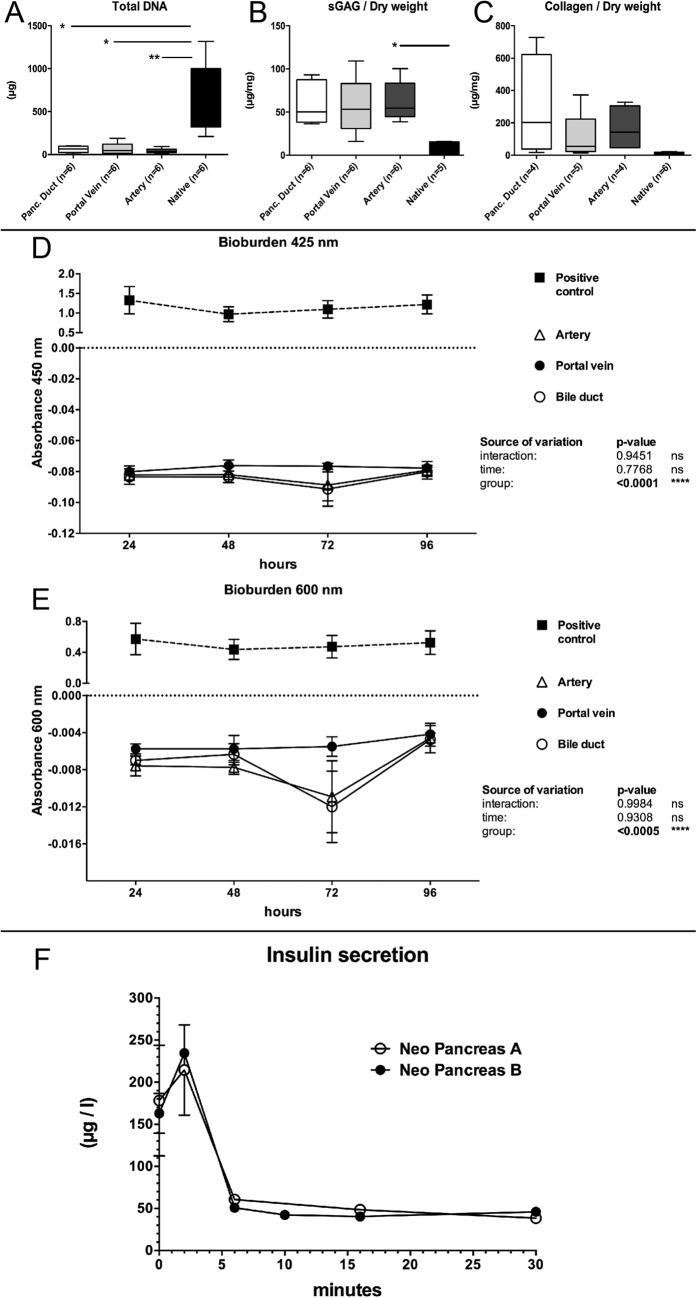
Biochemical evaluation. The amount of DNA significantly declined in all experimental groups compared to native controls (**A**). No statistically significant differences were observed between the experimental groups. The content of sulfated Glycosaminoglycans (sGAG) per dry weight (**B**) increased compared to native controls due to a loss of cellular protein mass. No statistically significant differences were observed between the experimental groups. The collagen content per dry weight (**C**) increased compared to native controls due to a loss of cellular protein mass. No statistically significant differences were observed between the experimental groups. Bioburden testing at 425 nm (**D**) and 600 nm (**E**) revealed that all experiments were sterile and not contaminated. Insulin analysis revealed that Insulin levels in the perfusate initially increased after high glucose perfusion and then were constant until the end of the perfusion (**F**).

In all three experimental groups, the total DNA content declined significantly compared with the controls (Arterial Perfusion: 44.24 ± 11.58 μg (p < 0.05); Portal Venous Perfusion: 67.49 ± 27.68 μg (p < 0.5); Pancreatic duct perfusion: 59.73 ± 15.89 μg (p < 0.5); C: 687.0 ± 168.3 μg (n = 6); Fig. 4A). No statistically significant differences between the three groups were found. The conservation of the main ECM components was confirmed via biochemical analysis showing remaining sGAG and hydroxyproline content in the matrix. A trend towards higher values could be observed for the sGAG content/dry weight in the experimental groups compared with the native controls (A: 62.14 ± 10.52 μg/mg (p < 0.5); PV: 56.26 ± 15.06 μg/mg; BD: 60.32 ± 25.58 μg/mg; C: 8.076 ± 4.081 μg/mg; Fig. 4B). However, the only significant difference was found between the arterial perfusion group and the native controls. The collagen content/dry weight slightly increased in the three groups with different perfusion routes compared with the native controls (A: 164.3 ± 70.67 μg/mg; PV: 108.9 ± 66.69 μg/mg; BD: 287.4 ± 159 μg/mg; C: 13.01 ± 2.573 μg/mg; Fig. 4C). However, no significant differences could be found between the groups.

### Contamination analysis of decellularized matrices

When a cell culture is contaminated with germs the culture medium becomes cloudy. Therefore, light absorbance levels of the culture medium was measured at 425 and 600 nm to analyze its turbidity and to quantify sterility of the decellularized matrices^25^. Bioburden analysis showed no change in the absorbance of any of the three experimental groups compared with the positive controls at both wavelengths throughout the experiment (Fig. 4D+E), indicating that the decellularized matrices remained sterile 96 hours after the process.

### Digital subtraction analysis of decellularized matrices

**Figure 5:**
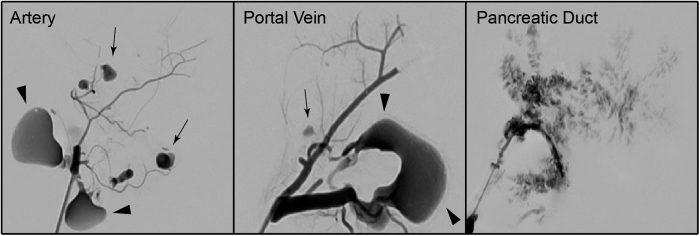
Digital subtraction angiography (DSA) of decellularized pancreata. DSA revealed a major difference between pancreata, which were decellularized via the Artey or the Portal Vein and the Pancreatic Duct: Contrast agent that was infused via the vascular system followed the conserved vascular protein network and leaked into the decellularized parenchymal space at the ends of the intraparenchymatous vessels (black arrows). When infused via the pancreatic duct, the contrast agent followed the ductular system, too, but leaked into the decellularized parenchymal space along all along the ductular system. Some contrast agent leaked through extra-pancreatic vessels outside the organs (black arrowheads).

The integrity of the vascular system within the decellularized pancreas was confirmed by angiography: Infusion of contrast agent via the arterial system and the portal vein showed an intact framework of the vessels with sparse emersion of contrast agent at the distal ends of the intra-pancreatic capillaries (Fig. 5). Angiography of the pancreatic duct showed a diffuse emersion of contrast agent along the ductular system into the parenchymal space (Fig. 5).

### Scanning electron microscopy (SEM) of decellularized matrices

**Figure 6:**
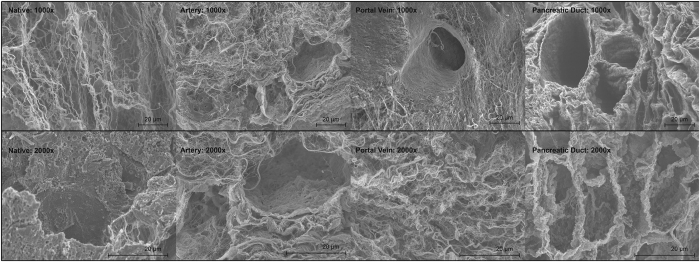
Scanning electron microscopy (SEM) of decellularized pancreata. SEM at a magnification of 1000 and 2000 was performed to analyze the matrix composition after decellularization via the different perfusion routes and compare them with native controls. SEM showed intact protein fibers of the decellularized pancreata and empty cellular spaces compared to native controls. No significant differences were observed between the three experimental groups.

A comparison of native and decellularized pancreata demonstrated the preservation of the three-dimensional microstructure after decellularization (Fig. 6). SEM images of decellularized pancreata showed the conservation of matrix components. In contrast to the native controls, empty spaces surrounded by networks consisting of fibers with different calibers can be found inside the ECM. Intact protein networks of the blood vessels could be confirmed by the appearance of the internal elastic lamina without any residual endothelial cells. In the examined specimens, no relevant differences between the experimental groups were observed.

### Evaluation of the repopulation process

**Figure 7:**
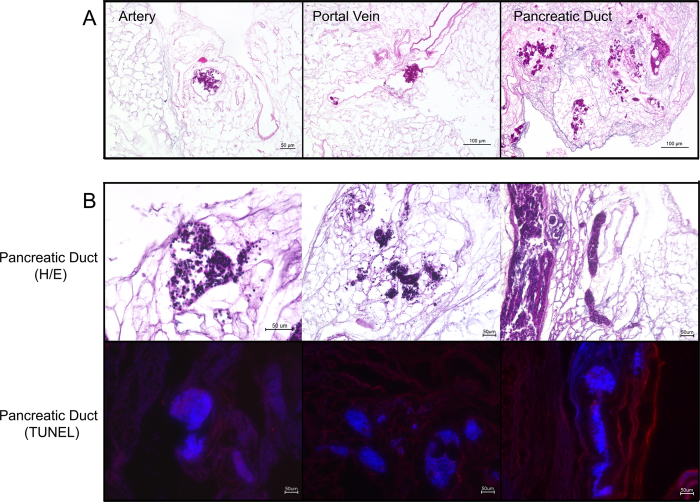
H/E and TUNEL staining of the repopulated pancreata. At first the optimal route for the repopulation process was evaluated (**A**): When infused via the vascular system either islets were stuck inside the protein network of the vessels or were flushed through the organ. When infused via the pancreatic duct, islets leaked into peri-ductular parenchymal space. More islets were found in the pancreas repopulated via the pancreatic duct. Therefore further repopulation experiments were performed via the pancreatic duct (**B**): H/E staining confirmed the previous observations and showed many intact islets inside the decellularized space. TUNEL staining (red) revealed that islets are TUNEL negative and DAPI positive (blue) and therefore viable after the process.

During repopulation, islets were observed inside the vascular or ductular network of the decellularized pancreas, depending on the infusion route. Islets followed the flow of the perfusate inside the network from proximal to distal into the periphery of the organ. Some islets then leaked from the visible vascular or ductular network and entered the decellularized parenchymal matrix, while others remained inside the decellularized vessels. Interestingly, islets infused via the pancreatic duct seemed to enter the parenchymal matrix faster and easier. This observation was confirmed by H/E staining of the repopulated matrices (Fig. 7): In total, more islets were found inside the organ, when infused via the pancreatic duct, compared to the aorta or the portal vein. Furthermore, more of these islets leaked into the parenchymal space. Since only a limited amount of islets and no other cells were infused, the majority of the decellularized space remained empty (Fig. 7).

Based on these observations two additional rat pancreata were repopulated via the pancreatic duct and used for *ex vivo* perfusion and glucose stimulated insulin secretion (GSIS) testing. In these experiments 17500 islets each were infused, using a multi-step recellularization protocol. Seeding efficiency of Experiment A and B was 81.96% and 84.5%, respectively.

### Viability and functionality of the Neo Pancreas grafts

At the beginning of the high glucose stimulation, insulin levels were 178 ± 66 μg/l and 163 ± 24 μg/l, respectively. After 2 minutes of high glucose perfusion insulin in the perfusate increased to the highest values at 215 ± 54 μg/l and 235 μg/l, respectively. Then, insulin constantly decreased and was 39 μg/l and 46 μg/l after 30 minutes of perfusion (Fig. 4F). Even after 90 minutes of perfusion, insulin was still detectable in the perfusate (22 μg/l and 40 μg/l, respectively; data not shown) indicating an insulin production of the islets during the whole experiment.

H/E and TUNEL staining confirmed that most of the islets remained intact after the manual infusion. Islets inside the decellularized pancreas matrix were negative for TUNEL staining, even after six hours of *ex vivo* perfusion for the GSIS testing, showing that islets remained viable after the process (Fig. 7).

## Discussion

The decellularization and recellularization of parenchymal organs has in recent years emerged in the field of tissue engineering. This interesting new technique could provide several improvements: by removing all cellular material from an allogeneic or even xenogeneic organ, a less immunogenic three-dimensional scaffold can be generated that can be repopulated with xenogeneic, allogeneic or autologous cells. If the endothelial integrity inside the vascular network is re-established by e.g. re- endothelialization^26^ the engineered organ could finally be implanted *in vivo*. Recent proof-of-concept studies showed promising *in-vivo* results for various recellularized organs such as the heart, lung, liver and kidney^27^,^28^,^29^,^30^ but available data on pancreas decellularization remain limited^1^,^20^,^21^,^22^,^23^.

We provide the first protocol for rat pancreas decellularization and recellularization with whole islets. To analyze the impact of the perfusion route, three different experimental groups, that were identical with regard to applied perfusates, flow rates and duration, were evaluated. Interestingly, in contrast to our own results in the liver^31^ and to the results of Goh *et al*.^21^, who stated that decellularization via the pancreatic duct was insufficient, in the presented study, the perfusion route only appeared to have a minor impact on decellularization efficiency. In none of the examined specimens any remaining cells were found and the microstructures of the decellularized organs, including immunohistochemical stainings of the important matrix components, were comparable.

The only major difference between the three experimental groups was revealed by angiography: While contrast agent given via the vascular system remained mainly inside the framework of the vascular system and only leaked in to parenchyma at the distal ends of the capillaries, contrast agent infused via the pancreatic duct followed the ductular system but spilled into the parenchymal space all along the ductular system. Thus, we hypothesized that the pancreatic duct could be the best perfusion route for repopulation of decellularized pancreas matrices: Islets represent units of different cells and are significantly larger than single cells^24^. If the basal lamina of the vascular network is still intact after decellularization, infused islets should not be able to pass through the vascular protein network into the decellularized parenchymal space. We infused islets through the arterial system or the portal venous system or the pancreatic duct and confirmed our angiographic observations: When given via the vascular system, a high amount of the islets either remained inside the vascular system and got stuck or islets were just flushed through the organ. In contrast, islets given via the pancreatic duct left the ductular system and were found inside the parenchyma. Interestingly, a recent publication on human pancreas decellularization showed a survival of islets cultured on slices of the decellularized pancreas in a perifusion chamber. However, whole decellularized pancreata were not repopulated with islets, possibly due to their size^22^.

In our study, functionality of the repopulated grafts was evaluated by *ex vivo* perfusion and glucose stimulated insulin secretion testing: Compared to physiological levels, we found relatively high glucose levels in the perfusate, which can be explained by a high number of islets used for the repopulation. Then, we found higher levels at the beginning of the perfusion with high glucose medium than we expected. This finding may result from different reasons: During the three hours of perfusion with low glucose medium we used a closed circuit to save perfusion medium. Then, after initiation of perfusion with high glucose the circuit was opened and fresh (high glucose) medium was inserted. Thus, secreted insulin might have accumulated in the circuit before initiation of high glucose perfusion. Furthermore, at least some islets will have been damaged during the process of repopulation and therefore some insulin might have been released from perished islets. However, we are able to show a clear increase of insulin after initiation of perfusion with high glucose medium and found constant insulin levels during the further perfusion even with the open circuit. These findings clearly show that islets are functional after the repopulation process, as confirmed by TUNEL staining after the perfusion experiments.

If the vascular (and ductular) protein network of the organ is conserved, the cellular integrity of these structures can also be re-assembled with (for example) endothelial cells, as shown by Orlando *et al*.^17^. Complete re-endothelialization will be essential because thrombosis will otherwise become a major issue after implantation^32^. In our study, the conservation of the vascular system was examined via macroscopic observation and confirmed in histological stainings, scanning electron microscopy and angiography. Thus, after parenchymal repopulation with islets, our next step will be the re-endothelialization of the vascular protein network.

However, we provide the first proof-of-concept that the parenchymal space of a decellularized pancreas can be repopulated with islets, despite their great size. We hypothesize that islets can leak into the parenchymal matrix through ruptures in the basal membrane of I) the conserved vascular protein network or even better II) through the protein network of the ductular system. In our experiments, repopulation via the pancreatic ductular system showed the most promising results. TUNEL staining and glucose stimulated insulin secretion revealed that islets remain viable and functional after the process.

## Conclusion

To the best of our knowledge, we herein present the first available protocol for whole rat pancreas decellularization and recellularization with islets of Langerhans and compare three perfusion routes (arterial, venous and ductular). We provide proof-of-concept that the repopulation of decellularized rat pancreata is possible via the pancreatic duct and that islets remain viable and functional after the process. The presented technique can serve as a bioengineering platform for further recellularization and implantation studies.

## Methods

### Animals

All animal work was performed in accordance with local law and approved by the State Office of Health and Local Affairs (LAGeSo, Berlin, Germany; Reg. No. T0139/13, O0262/13 and O264/13).

Forty-four male and female F344 DPP IV-, Wistar and Lewis eGFP rats (FEM, Charité, Berlin, Germany) weighing between 150 and 400 g were used for the decellularization experiments.

Fifteen female outbred Wistar rats (Harlan Sprague Dawley Inc., Indianapolis, IN, USA) weighing between 300 and 400 g were used for the islet isolation procedure.

Sterile conditions were maintained throughout all surgical procedures.

### Rat Pancreas Harvesting

Rats were anaesthetized via the inhalation of isoflurane (abbvie, North Chicago, IL, USA) (3.5% for induction/2% for maintenance) and administered a subcutaneous injection of ketamine (Pfizer, New York City, NY, USA) (10 mg/kg), medetomidine hydrochloride (cp pharma, Burgdorf, Germany) (0.1 mg/kg) and metamizol (ratiopharm, Burgdorf, Germany) (100 mg/kg).

For perfusion via the portal vein (PV) and the abdominal aorta (A), the protocol for donor pancreatectomy published by Klempnauer and Settje^33^ was modified. We cannulated the designated vessel after injecting 500 IE of heparin (Rotexmedia, Trittau, Germany) into the inferior vena cava. The portal vein was cannulated retrogradely with a shortened 17 G catheter (Becton, Dickinson and Company, Franklin Lakes, NJ, USA). The aorta was cannulated retrogradely with a 17 G catheter after preparation of all branches. For the perfusion via the pancreatic duct (PD), the major duodenal papilla was exposed and ligated once with Silk 5–0. A small incision was made at common bile duct and a shortened 24 G catheter (Becton, Dickinson and Company) was inserted anterogradely.

All explanted organs were placed in a 100 ml beaker filled with cold Ringer-Lactate buffer and directly used for decellularization (PV, A and PD), or direct preparation for further analyses (Controls).

### Perfusion Decellularization

To avoid contamination, the complete procedure was performed under sterile conditions in a laminar airflow cabinet (Heraeus Instruments, Hanau, Germany). The cannulated pancreata were connected to a perfusion system and 1% Triton X100 (Carl Roth, Karlsruhe, Germany) was perfused at 10 ml/min. for 60 minutes through the portal vein. Next, 0.5% sodium dodecyl sulfate (SDS) (Carl Roth) was used as a perfusate for 120 minutes. Again 1% Triton X-100 was used to perfuse the organs for 15 minutes at 10 ml/min. Then, phosphate-buffered saline solution pH 7.4 (PBS) (Biochrom, Berlin, Germany) was rinsed through the organs at 2 ml/min. for 4 hours.

### Islet isolation

Islet isolation was performed as described by Schubert *et al*.^34^. After euthanasia, a digestion solution containing Collagenase (Sigma-Aldrich, St. Louis, MO, USA) and DNase I (F. Hoffmann-La Roche, Basel, Switzerland) was injected anterogradely into the common bile duct. The pancreata were digested for 15 min., islets were isolated with a discontinuous Ficoll (Sigma-Aldrich) gradient and cultured in RPMI 1640 (Gibco, ThermoScientific, Venlo, Netherlands) supplemented with 10% (vol/vol) FBS (Biochrom). Purity and the amount of islets were determined using dithizone staining (Sigma-Aldrich).

### Recellularization

At first recellularization was performed to test the best perfusion route: Isolated islets were reseeded on the decellularized matrix via a single-step perfusion technique. Approximately 2,000 islets in 3 ml islet medium each were manually infused via the portal vein or the aorta or the pancreatic duct. The repopulation process was performed under a M651 surgical microscope (Leica Microsystems, Wetzlar, Germany) and filmed with a MC170HD microscope camera (Leica Microsystems).

Further pancreata were recellularized with a modified technique via the pancreatic duct to test viability and functionality of the islets after the process: Decellularized pancreata were perfused with PBS 1 ml/min. overnight at room temperature. One hour before recellularization the organs were transferred into a petri dish filled with islet medium and incubated at 37 °C (95% O_2_/5% CO_2_). Recellularization was conducted using a 3-step manual infusion technique. At first, all cannulated outflows of the decellularized pancreas matrices were closed and 5 ml islet medium was infused via the extrahepatic bile duct. In total 6 ml islet medium with approximately 17,500 islets was infused via the same route consisting of 2 min. infusion periods followed by 5 min. of static seeding.

### *Ex-vivo* Perfusion and Glucose stimulated Insulin Secretion (GSIS)

**Figure 8:**
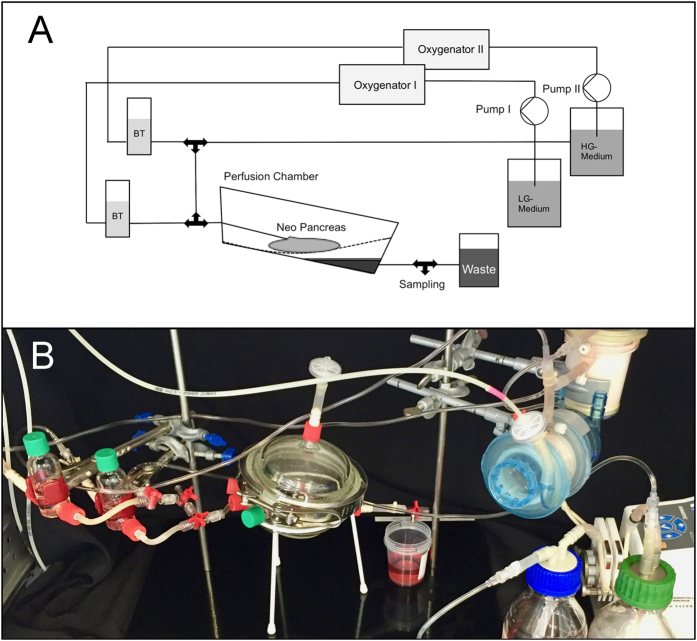
Scheme and photograph of the bioreactor for *ex vivo* perfusion. The reactor consisted of a perfusion chamber and two separate circuits for low and high glucose medium, each consisting of a pump, an oxygenator and a bubble trap. Two circuits were used to constantly circulate and oxygenate the perfusion medium and to ensure a fast switch from low to high glucose perfusion.

After repopulation, the recellularized organs were connected to the perfusion reactor, kept at 37 °C (Fig. 8) but were not perfused for one hour to permit adherence of the islets inside the matrix. Then, the organs were perfused for 180 min. with low glucose islet medium (4 mmol/l) followed by a 90 min. perfusion with high glucose islet medium (20 mmol/l). Perfusion was performed via the cannulated arterial system. Perfusate freely flew out of the pancreas into the perfusion chamber. Samples from the effluent inside the perfusion chamber were taken using a syringe at different time points and stored at −20 °C before further analyses (Fig. 4F). Seeding efficiency was calculated by dithizone staining of the remaining islets inside the petri dish and inside the reactor at the end of the adherence time.

### Histological evaluation

Paraformaldehyde-fixed (Herbeta Arzneimittel, Berlin, Germany) and paraffin-embedded (Sigma-Aldrich) tissue sections were cut 5-μm-thick and stained with Hematoxylin and Eosin (AppliChem, Darmstadt, Germany), Alcian Blue (Morphisto, Frankfurt am Main, Germany) and Sirius Red (Sigma-Aldrich).

The immunohistochemistry staining for Collagen IV and Laminin used a 3% hydrogen peroxide solution, antigen retrieval with a 0.01 M citrate buffer (Agilent Technologies, Glostrup, Denmark) and 3% goat serum for blocking (Agilent Technologies). The sections were then incubated 1:400 with a rabbit anti-mouse polyclonal collagen IV antibody (Abcam, Cambridge, United Kingdom, Cat #ab6586) or 1:50 with a rabbit anti-mouse polyclonal laminin antibody (Abcam, Cat #ab11575) for 1 hour at 37 °C. The secondary goat anti-rabbit HRP antibody (Abcam, Cat #ab6721) was diluted 1:400 and incubated for 30 minutes followed by visualization with 3,3′-Diaminobenzidine (Agilent Technologies). Fibronectin staining included antigen retrieval with a 0.01 M citrate buffer, peroxidase block (Agilent Technologies) and protein block (Agilent Technologies). Sections were then incubated 1:100 with a rabbit polyclonal fibronectin antibody (Abcam, Cat #ab23751) at room temperature overnight. LSAB System-HRP (Agilent Technologies) was used according manufacturer ´s instructions and 3,3′-Diaminobenzidine as substrate for visualization. Finally, all sections were counterstained with Mayer ´s hematoxylin and mounted with Aquatex (Merck, Darmstadt, Germany).

To detect apoptosis inside the repopulated pancreas grafts we performed terminal deoxynucleotidyl transferase dUTP nick end labeling (TUNEL) staining using an *In-Situ* Cell Death Detection Kit, Fluorescein (F. Hoffmann-La Roche). To stain the tissue we used the labeling protocol for difficult tissue provided by the manufacturer. Afterwards the tissue was counterstained with DAPI (Sigma-Aldrich).

Images were recorded with a BZ-9000 BIOREVO microscope (Keyence, Osaka, Japan).

### Biochemical analysis

For biochemical analysis, diethylpyrocarbonate (DEPC) (AppliChem) was added to the samples (n = 6 for each group), and they were homogenized with an Ultra-Turrax T25 (Janke & Kunkel IKA Labortechnik, Staufen, Germany). For DNA quantification, the DNeasy Blood & Tissue Kit (Qiagen, Venlo, Netherlands) was used to purify the DNA according to the protocol for small sample sizes provided by the manufacturer. The DNA content was measured using a NanoDrop 2000 C UV-Vis Spectrophotometer (ThermoScientific, Venlo, Netherlands) with a pre-configuration for DNA at 260 nm. For hydroxyproline detection, the Total Collagen Assay (QuickZyme, Leiden, Netherlands) was used following the instructions of the manufacturer. The hydroxyproline content was measured at an absorbance maximum of 570 nm with a FluoStar Optima (BMG Labtech, Ortenberg, Germany) and run against a standard curve (6–300 μg/ml). The sGAG content was determined using a method described by Farndale *et al*.^35^. 1 ml of a 1,9-Dimethylmethylene blue (Sigma-Aldrich) color reagent was mixed with the same amount of sample solution that had been incubated in a buffer containing papain and run directly at 525 nm on the NanoDrop 2000 C. A standard curve (0–200 μg/ml) was obtained using Chondroitin-4-Sulphate (Carl Roth) with the same method.

A rat insulin ELISA (Mercodia AB, Uppsala, Sweden) was used to measure insulin levels in the perfusate. The protocol was performed according to the protocol provided by the manufacturer.

### Bioburden analysis

To check for bacterial contamination, 5 ml of the perfusion perfusate was mixed with 7 ml SOC medium in a partially opened 15 ml Falcon tube. The mixture was incubated at 37 °C and absorbance at 425 and 600 nm was run against blank SOC medium with PBS after 24, 48, 72 and 96 hours using the NanoDrop 2000 C.

### Digital Subtraction Angiography

To visualize ductal and vascular anatomy, digital subtraction angiography was performed in cooperation with the department of Radiology. A 1:1 mixture of Lipiodol (Guerbet, Villepinte, France) and Histoacryl (B. Braun, Melsungen, Germany) was used as a contrast agent. Images were acquired with an Allura Xper FD20/20 (Philips Medical Systems, Hamburg, Germany) angiography unit. After flushing with 10% glucose solution, the contrast agent was injected manually into the cannulated portal vein, artery and pancreatic duct under fluoroscopic control.

### Scanning electron microscopy (SEM)

Scanning electron microscopy was performed by the electron microscopy core facility at the Charité Universitätsmedizin Berlin. The samples were fixated with 2.5% glutaraldehyde in 0.1 M sodium cacodylate buffer, rinsed, post-fixated with 1% osmium tetroxide and rinsed again. Then, the specimens were dehydrated with an ascending row of ethanol and critical-point-dried in a Polaron E3000 (Quorum Technologies, Laughton, United Kingdom). The samples were mounted on an aluminum pin and sputter-coated with a gold/palladium alloy to generate conductivity with a MED 020 (BAL-TEC, Balzers, Liechtenstein).

Pictures were taken with a DSM 982 Gemini (Zeiss, Oberkochen, Germany).

### Statistical analysis

Statistical analysis and visualization were performed using Prism 6.0 g for Mac OS X (GraphPad Software Inc., La Jolla, CA, USA). Numeric data were expressed as the means ± standard error of means (SEM). Because a normal distribution could not be assumed due to the small sample size, group comparisons were performed using the Kruskal-Wallis test followed by Dunn ´s multiple comparison analysis. Probability values p < 0.05 were regarded as statistically significant.

## Additional Information

**How to cite this article**: Napierala, H. *et al*. Engineering an endocrine Neo-Pancreas by repopulation of a decellularized rat pancreas with islets of Langerhans. *Sci. Rep.* **7**, 41777; doi: 10.1038/srep41777 (2017).

**Publisher's note:** Springer Nature remains neutral with regard to jurisdictional claims in published maps and institutional affiliations.

## Figures and Tables

**Figure 1:**
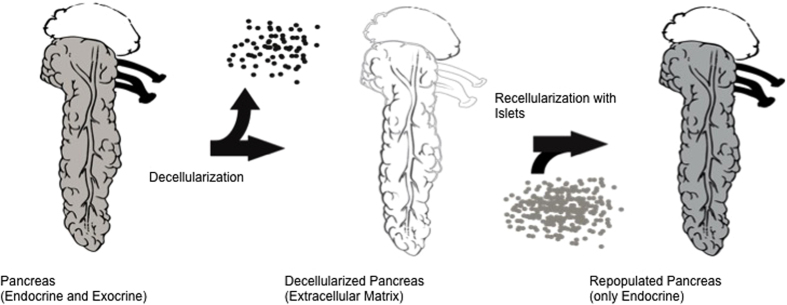
Concept to generate an Endocrine Neo-Pancreas. A (xenogene) pancreas is decellularized by perfusion with alkaline detergents. The resulting non-immunogenic matrix conserves the organ specific extracellular matrix including the vascular and ductular protein network. The matrix is then repopulated by infusion of islets and endothelial cells to generate a functional and implantable organ.

## References

[b1] Mirmalek-Sani S. H. . Porcine pancreas extracellular matrix as a platform for endocrine pancreas bioengineering. Biomaterials 34, 5488–5495 (2013). 23583038 10.1016/j.biomaterials.2013.03.054 PMC3680884

[b2] Imperatore G. . Projections of Type 1 and Type 2 Diabetes Burden in the U.S. Population Aged < 20 Years Through 2050: dynamic modeling of incidence, mortality and population growth. Diabetes Care 35, 2515–2520 (2012). 23173134 10.2337/dc12-0669 PMC3507562

[b3] Frier B. M. Hypoglycaemia in diabetes mellitus: epidemiology and clinical implications. Nat Rev Endocrinol 10, 711–722 (2014). 25287289 10.1038/nrendo.2014.170

[b4] Atkinson M. A., Eisenbarth G. S. & Michels A. W. Type 1 diabetes. Lancet 383, 69–82 (2014). 23890997 10.1016/S0140-6736(13)60591-7 PMC4380133

[b5] Barton F. B. . Improvement in outcomes of clinical islet transplantation: 1999–2010. Diabetes Care 35, 1436–1445 (2012). 22723582 10.2337/dc12-0063 PMC3379615

[b6] Ludwig B., Ludwig S., Steffen A., Saeger H. D. & Bornstein S. R. Islet versus pancreas transplantation in type 1 diabetes: competitive or complementary? Curr Diab Rep 10, 506–511 (2010). 20830612 10.1007/s11892-010-0146-y

[b7] Balzano G.. Autologous islet transplantation in patients requiring pancreatectomy: a broader spectrum of indications beyond chronic pancreatitis. Am J Transplant, doi: 10.1111/ajt.13656 (2015 Dec 22) [Epub ahead of print]. 26695701

[b8] Olsson R., Olerud J., Pettersson U. & Carlsson P. O. Increased numbers of low-oxygenated pancreatic islets after intraportal islet transplantation. Diabetes 60, 2350–2353 (2011). 21788575 10.2337/db09-0490 PMC3161309

[b9] Kawahara T., Kin T. & Shapiro A. M. A comparison of islet autotransplantation with allotransplantation and factors elevating acute portal pressure in clinical islet transplantation. J. Hepato-Biliary-Pancreat. Sci. 19, 281–288 (2012). 10.1007/s00534-011-0441-2 21879320

[b10] Kawahara T. . Portal vein thrombosis is a potentially preventable complication in clinical islet transplantation. Am J Transplant 11, 2700–2707 (2011). 21883914 10.1111/j.1600-6143.2011.03717.x PMC3226916

[b11] Naziruddin B. . Evidence for instant blood-mediated inflammatory reaction in clinical autologous islet transplantation. Am J Transplant 14, 428–437 (2014). 24447621 10.1111/ajt.12558

[b12] Struecker B., Raschzok N. & Sauer I. M. Liver support strategies: cutting- edge technologies. Nat. Rev. Gastroenterol. Hepatol. 11, 166–176 (2014). 24166083 10.1038/nrgastro.2013.204

[b13] Song J. J. & Ott H. C. Organ engineering based on decellularized matrix scaffolds. Trends Mol. Med. 17, 424–432 (2011). 21514224 10.1016/j.molmed.2011.03.005

[b14] Orlando G. . Regenerative medicine as applied to solid organ transplantation: current status and future challenges. Transpl. Int. 24, 223–232 (2011). 21062367 10.1111/j.1432-2277.2010.01182.x PMC3817209

[b15] Badylak S. F., Weiss D. J., Caplan A. & Macchiarini P. Engineered whole organs and complex tissues. Lancet 379, 943–952 (2012). 22405797 10.1016/S0140-6736(12)60073-7

[b16] Sullivan D. C. . Decellularization methods of porcine kidneys for whole organ engineering using a high-throughput system. Biomaterials 33, 7756–7764 (2012). 22841923 10.1016/j.biomaterials.2012.07.023

[b17] Orlando G. . Production and implantation of renal extracellular matrix scaffolds from porcine kidneys as a platform for renal bioengineering investigations. Ann Surg 256, 363–370 (2012). 22691371 10.1097/SLA.0b013e31825a02ab

[b18] Wang Y. . Lineage restriction of human hepatic stem cells to mature fates is made efficient by tissue-specific biomatrix scaffolds. Hepatology 53, 293–305 (2011). 21254177 10.1002/hep.24012

[b19] Zhu S. . Human pancreatic beta-like cells converted from fibroblasts. Nat. Commun. 7, 10080 (2016). 26733021 10.1038/ncomms10080 PMC4729817

[b20] De Carlo E. . Pancreatic acellular matrix supports islet survival and function in a synthetic tubular device: *in vitro* and *in vivo* studies. INT J MOL MED 25, 195–202 (2010). 20043127

[b21] Goh S. K. . Perfusion-decellularized pancreas as a natural 3D scaffold for pancreatic tissue and whole organ engineering. Biomaterials 34, 6760–6772 (2013). 23787110 10.1016/j.biomaterials.2013.05.066 PMC3748589

[b22] Peloso A. . The Human Pancreas as a Source of Protolerogenic Extracellular Matrix Scaffold for a New-generation Bioartificial Endocrine Pancreas. Ann Surg, doi: 10.1097/sla.0000000000001364 (2015 Nov 26) [Epub ahead of print]. PMC4882269 26649588

[b23] Wu D. . 3D Culture of MIN-6 Cells on Decellularized Pancreatic Scaffold: *In Vitro* and *In Vivo* Study. Biomed Res Int 2015 432645 (2015). 10.1155/2015/432645 PMC4672115 26688810

[b24] von Mach M. A. . Size of pancreatic islets of Langerhans: a key parameter for viability after cryopreservation. Acta Diabetol 40, 123–129 (2003). 14605968 10.1007/s00592-003-0100-4

[b25] Guyette J. P., Gilpin S. E., Charest J. M., Tapias L. F., Ren X. & Ott H. C. Perfusion decellularization of whole organs. Nat. Prot. 9, 1451–1468 (2014). 10.1038/nprot.2014.097 24874812

[b26] Crapo P. M., Gilbert T. W. & Badylak S. F. An overview of tissue and whole organ decellularization processes. Biomaterials 32, 3233–3243 (2011). 21296410 10.1016/j.biomaterials.2011.01.057 PMC3084613

[b27] Ott H. C. . Perfusion-decellularized matrix: using nature’s platform to engineer a bioartificial heart. Nat. Med. 14, 213–221 (2008). 18193059 10.1038/nm1684

[b28] Ott H. C. . Regeneration and orthotopic transplantation of a bioartificial lung. Nat. Med. 16, 927–933 (2010). 20628374 10.1038/nm.2193

[b29] Song J. J. . Regeneration and experimental orthotopic transplantation of a bioengineered kidney. Nat. Med. 19, 646–651 (2013). 23584091 10.1038/nm.3154 PMC3650107

[b30] Uygun B. E. . Organ reengineering through development of a transplantable recellularized liver graft using decellularized liver matrix. Nat. Med. 16, 814–820 (2010). 20543851 10.1038/nm.2170 PMC2930603

[b31] Struecker B. . Improved rat liver decellularization by arterial perfusion under oscillating pressure conditions. J Tissue Eng Regen Med, doi: 10.1002/term.1948 (2014 Sep 4) [Epub ahead of print]. 25185781

[b32] Jiang B., Akgun B., Lam R. C., Ameer G. A. & Wertheim J. A. A polymer- extracellular matrix composite with improved thromboresistance and recellularization properties. Acta biomater. 18, 50–58 (2015). 25712388 10.1016/j.actbio.2015.02.015 PMC4395555

[b33] Klempnauer J. & Settje A. Vascularized pancreas transplantation in the rat–details of the microsurgical techniques, results, and complications. Transpl. Int. 2, 84–91 (1989). 2789669 10.1007/BF02459325

[b34] Schubert U. . Transplantation of pancreatic islets to adrenal gland is promoted by agonists of growth-hormone-releasing hormone. Proc. Natl. Acad. Sci. USA 110, 2288–2293 (2013). 23345449 10.1073/pnas.1221505110 PMC3568317

[b35] Farndale R. W., Buttle D. J. & Barrett A. J. Improved quantitation and discrimination of sulphated glycosaminoglycans by use of dimethylmethylene blue. Biochim. Biophys. Acta 883, 173–177 (1986). 3091074 10.1016/0304-4165(86)90306-5

